# Curcumin-In-Deformable Liposomes-In-Chitosan-Hydrogel as a Novel Wound Dressing

**DOI:** 10.3390/pharmaceutics12010008

**Published:** 2019-12-20

**Authors:** Selenia Ternullo, Laura Victoria Schulte Werning, Ann Mari Holsæter, Nataša Škalko-Basnet

**Affiliations:** Drug Transport and Delivery Research Group, Department of Pharmacy, University of Tromsø The Arctic University of Norway, Universitetsveien 57, 9037 Tromsø, Norway; selenia89.ternullo@gmail.com (S.T.); laura.v.schulte-werning@uit.no (L.V.S.W.); ann-mari.holsater@uit.no (A.M.H.)

**Keywords:** curcumin, deformable liposomes, liposome surface charge, hydrogel, chitosan, wound therapy

## Abstract

A liposomes-in-hydrogel system as an advanced wound dressing for dermal delivery of curcumin was proposed for improved chronic wound therapy. Curcumin, a multitargeting poorly soluble active substance with known beneficial properties for improved wound healing, was incorporated in deformable liposomes to overcome its poor solubility. Chitosan hydrogel served as a vehicle providing superior wound healing properties. The novel system should assure sustained skin delivery of curcumin, and increase its retention at the skin site, utilizing both curcumin and chitosan to improve the therapy outcome. To optimize the properties of the formulation and determine the effect of the liposomal charge on the hydrogel properties, curcumin-containing deformable liposomes (DLs) with neutral (NDLs), cationic (CDLs), and anionic (ADLs) surface properties were incorporated in chitosan hydrogel. The charged DLs affected the hydrogel’s hardness, cohesiveness, and adhesiveness. Importantly, the incorporation of DLs, regardless of their surface charge, in chitosan hydrogel did not decrease the system’s bioadhesion to human skin. Stability testing revealed that the incorporation of CDLs in hydrogel preserved hydrogel´s bioadhesiveness to a higher degree than both NDLs and ADLs. In addition, CDLs-in-hydrogel enabled the most sustained skin penetration of curcumin. The proposed formulation should be further evaluated in a chronic wound model.

## 1. Introduction

The treatment of chronic wounds is one of the most important global health care issues in the aging society. Chronic wounds are challenging to treat due to the diversity of factors contributing to slow/impaired healing. The hostile environment of the wound comprising degradative enzymes and elevated pH, as well as the complexity of physiological processes, requires an effective advanced drug delivery system as a wound dressing [1,2]. An ideal wound dressing should reduce infection, moisturize the wound, stimulate the healing mechanism, accelerate the wound closure, and reduce/control scar formation [3].

Recently, the incessant release of free radicals has been proposed as one of the key factors responsible for activation of the inflammatory system, leading to impaired repair of the wound [4]. Therefore, the effective control of free radical levels in the wound and subsequent inflammation could assist in enhanced wound healing and scar control. Various antioxidants could be utilized to control free radicals during wound healing.

Curcumin has been proposed as one of the most promising [5]. Moreover, curcumin is a molecule able to active multiple pathways, contributing to improved wound healing [6]. The antioxidant, anti-inflammatory, and anti-bacterial properties of this pleiotropic molecule need to be more utilized [7,8]. Although curcumin is a very promising molecule to be included in wound dressings, its hydrophobicity, extensive metabolism, and limited skin penetration often hamper its wider use [9]. We have recently proven that curcumin, a multitargeting active substance, exhibits superior anti-inflammatory properties when incorporated in tailored deformable liposomes [10]. In this work, we went a step further in the development of curcumin-based wound dressings and combined the beneficial properties of curcumin as an active moiety and chitosan hydrogel as a superior vehicle to form a bioactive wound dressing targeting the treatment of chronic wounds.

An ideal dressing should assure an enhanced therapeutic outcome and reduction of pain accompanied with wound dressing changes [11]. Among the possible material to be used in the manufacturing of bioactive dressings, natural polymer-based wound dressings, such as hydrogels, represent promising advanced delivery systems with a low risk of toxicity and side effects due to their biodegradability and biocompatibility [12,13]. Hydrogels mimic the biochemical, biomechanical, and structural features of the extracellular matrix (ECM), serving as superior matrices for wound treatment [13,14]. Hydrogels additionally possess good bioadhesiveness that can contribute to a prolonged retention time of the incorporated drug/active substance at the wounded skin site. This, together with the sustained drug release via hydrogel, can result in higher drug/active substances’ concentrations at the skin site while reducing possible systemic absorption and consequent adverse effects [15].

Chitosan represents one of the most studied natural polymers for the development of effective hydrogel-based wound dressings due to its numerous intrinsic biological properties. Its biocompatibility and bioadhesiveness, together with intrinsic bacteriostatic effect, make this biopolymer effective in promoting wound healing while also preventing biofilm formation [16,17]. In spite of the numerous advantages of chitosan hydrogels, the hydrophilic environment provided by chitosan hydrogel limits the incorporation of lipophilic drugs/active compounds and remains a pharmaceutical challenge.

Liposomes as carriers for poorly soluble drugs/active molecules assist in overcoming this hydrogel limitation; the combination of these two delivery systems is a promising approach to achieve controlled dermal drug delivery and effective localized skin therapy [18,19,20]. The liposomal phospholipid bilayers allow the incorporation of lipophilic substances, improving their solubilization and enabling their inclusion into the hydrophilic chitosan hydrogel. Moreover, the ability of liposomes to assure sustained drug/substance release might improve targeted drug delivery to the specific skin layer(s) [19,21]. Liposomes also benefit from their incorporation in the hydrogel vehicle. The hydrogel can prevent rapid liposome clearance from the skin site and additionally protect from rapid degradation by preserving their membrane integrity [22]. Moreover, incorporation of liposomes in hydrogel will confer adequate viscosity to the liposomal dispersions to be topically administered [23].

The drug/substance release from a liposomes-in-hydrogel system is determined by the hydrogel properties, such as the polymer composition, mesh size, and porosity, combined with the liposomal physicochemical characteristics, namely the liposomal composition, size, and surface charge. In addition, the lipophilicity of the incorporated drug needs to be considered [24]. To the best of our knowledge, relatively little has been published on the incorporation of deformable liposomes (DLs) in chitosan hydrogel. DLs have shown superior ability in promoting drug penetration into the deeper skin layers in comparison to conventional liposomes [10,25]. In this study, we designed the DLs-in-chitosan hydrogel as a combined advanced delivery system, targeting the treatment of chronic wounds. Considering that chitosan has lateral amino groups conferring positive charge to its chains, the incorporation of charged liposomes could trigger interactions between the two delivery systems, affecting the hydrogel’s texture properties, liposomes’ integrity, and drug release [24]. We therefore focused on the effect of the liposomal surface charge on the hydrogel’s properties, such as the texture properties and bioadhesiveness; neutral (NDLs), cationic (CDLs), and anionic (ADLs) DLs were individually incorporated in chitosan hydrogel.

The effect of the liposomal surface charge on curcumin penetration from DLs-in-hydrogel systems through the ex vivo full thickness human skin was also evaluated.

## 2. Materials and Methods

### 2.1. Materials

Curcumin (≥94% curcuminoid content; ≥80% curcumin) was purchased from Sigma-Aldrich (St. Louis, MO, USA). Lipoid S 100 (>94% soybean phosphatidylcholine, PC) was a generous gift from Lipoid GmbH (Ludwigshafen, Germany). High molecular weight (MW) chitosan (Brookfield viscosity 800.000 cps and degree of deacetylation of 77%), polysorbate 20, stearylamine (SA), sodium deoxycholate (SDCh), methanol, disodium hydrogen phosphate dihydrate, monobasic potassium phosphate, sodium chloride, propylene glycol (PG), glycerol, and acetic acid were all purchased from Sigma-Aldrich (St. Louis, MO, USA). Albunorm^®^ (human serum albumin, 200 mg/mL) was produced by Octapharma AG (Lachen, Switzerland).

### 2.2. Preparation and Characterization of Deformable Liposomes

Curcumin-containing DLs bearing different surface charges were prepared by the film hydration method as previously described [26]. NDLs comprised PC and polysorbate 20 (total 200 mg), in a weight ratio of 85:15. CDLs comprised the same lipid and surfactant composition as NDLs, in addition to SA (weight ratio to PC 1:9). ADLs were composed of PC and SDCh (total 200 mg) in a ratio of 85:15 (*w*/*w*) (Table 1). All DLs were prepared by dissolving curcumin (20 mg), PC, and, when applicable, the different surfactants, in methanol. The organic solvent was evaporated under vacuum (55 mbar) at 55 °C (Büchi Rotavapor R-124 with Büchi Vacuum Pump V-700, Büchi Labortechnik AG, Flawil, Switzerland) and the obtained film was hydrated with 10 mL of phosphate buffer saline (PBS). PBS (pH 7.4) contained the following salts composition: 2.98 g/L Na_2_HPO_4_·2H_2_O, 0.19 g/L KH_2_PO_4_, 8 g/L NaCl. The liposomal dispersions were kept at 4 °C for 24 h prior to size reduction.

All DLs were reduced to a vesicle size of 200 × 300 nm by hand extrusion through the polycarbonate membrane (Nuclepore^®^ Track-Etched Membranes, Whatman House, Maidstone, UK). In brief, all DLs were extruded five times through 800-nm pore size membrane. NDLs were further extruded four times through 400-nm pore size membrane, whereas CDLs and ADLs were extruded through the same pore size membrane two and seven times, respectively. All liposomal dispersions were characterized in terms of vesicle size by photon correlation spectroscopy using a NICOMP Submicron Particle Sizer Model 370 (NICOMP Particle Sizing system, Santa Barbara, CA, USA) and zeta potential using Malvern Zetasizer Nano—ZS (Malvern, Oxford, UK) [26].

The entrapment efficiency of curcumin in DLs was determined after the centrifugation of the liposomal dispersion at 3000 *g* for 10 min using a Biofuge stratos centrifuge (Heraeus instruments GmbH, Hanau, Germany) to remove unentrapped curcumin. Liposomes were subsequently dissolved in methanol and the entrapped curcumin determined spectophotometrically at 425 nm (SpectraMax 190 Microplate Reader, Molecular Devices, San Jose, CA, USA). The entrapment efficiency was expressed as curcumin/lipid ratio (µg/mg), after quantification of the PC content in all DLs formulations (data not shown).

### 2.3. Preparation of Chitosan Hydrogels

Chitosan hydrogels were prepared according to a method described earlier [27]. In short, glycerol (10%, *w*/*w*) was mixed with an aqueous solution of acetic acid (2.5%, *w*/*w*). High MW chitosan (2.5%, *w*/*w*) was introduced into the mixture by hand stirring. To remove entrapped air, the mixture was bath-sonicated for 30 min. The prepared hydrogels were stored in a sealed container at room temperature for 48 h to allow complete swelling.

### 2.4. Preparation of DLs-In-Hydrogels

To prepare 10 g of liposomes-in-hydrogel formulation, 1.5 g of the different DLs (15%, *w*/*w*) were individually incorporated in the freshly prepared chitosan hydrogel after it was allowed to swell for 48 h at room temperature (Section 2.3) by hand-stirring [28]. The final concentration of curcumin in the liposomal hydrogel was 135 µg/g. As a control, free curcumin was incorporated in the hydrogel in the same concentration after dissolving in PG and subsequent incorporation in chitosan hydrogel by hand stirring. The DLs-in-hydrogel formulations were kept at room temperature for 2 h prior to further handling. The homogeneity test was performed to assure that liposomes were well dispersed within the liposomes-in-hydrogel formulation by sampling (5 aliquots were sampled from various places within hydrogel) and the curcumin concentration was determined in each aliquot.

### 2.5. Texture Analysis

All liposomes-in-hydrogel formulations were characterized in terms of the hardness, cohesiveness, and adhesiveness using a Texture Analyzer TA.XT Plus (Stable Micro Systems Ltd., Surrey, UK) [27]. The hydrogel (25 g) was placed in a beaker and a disc (35 mm in diameter) pushed down into the gel (distance of 10 mm) and withdrawn at a speed of 4 mm/s. The starting position of the disc was below the hydrogel surface. The hardness was calculated as the maximal force achieved when the disc was pushed down into the gel. The cohesiveness was expressed as the work necessary to push down the disc into the hydrogel while the adhesiveness as the work required during the upwards movement of the disc. Each formulation was tested five times and the measurements performed in triplicates.

### 2.6. Bioadhesion Test

The bioadhesion of hydrogels to the skin was tested as described earlier [28]. The full thickness human skin originated from the abdomen of female patients after plastic surgery. A written consent was obtained from the patients prior the surgery. No ethical approval was required from the Norwegian Ethical Committee, since we used skin to be disposed of after the surgery; the experiments were conducted according to the Declaration of Helsinki principles. The skin was cleared from the subcutaneous fatty tissue and rinsed with PBS prior the storage at −20 °C. The skin was thawed in PBS ca. 30 min prior to the experiment and subsequently fixed on a rig for Texture Analyzer TA.XT Plus (Stable micro systems, Surrey, UK). The bioadhesion test was performed by filling a die with the hydrogel formulation (150 µL), which was consequently pinched onto the skin for 10 s applying a force of 25 g. The bioadhesion was determined by measuring the force required to detach the die from the skin (detachment speed of 0.1 mm/s). Moreover, the die was weighed before and after each bioadhesion test to determine the amount of formulation retained onto the skin surface at the end of the test. Each formulation was tested five times and the human skin rinsed with ethanol between each measurement. Tests were conducted in triplicates.

### 2.7. Hydrogel Stability Testing

To evaluate the chitosan hydrogel stability and the influences of the liposomal surface charge on the stability of DLs-in-hydrogel, the stability testing was performed after the storage of the DLs-in-chitosan hydrogels for one month at room temperature (23–24 °C). The texture properties (Section 2.5) and bioadhesiveness (Section 2.6) of the chitosan hydrogel were determined before (one day after the formulation was prepared) and after the storage (day 30).

### 2.8. Ex Vivo Skin Penetration Studies

Ex vivo curcumin penetration from the different DLs-in-chitosan hydrogels was evaluated using the full thickness human skin in Franz diffusion cells (diffusion area of 1.77 cm^2^, PermeGear, Bethlehem, PA, USA) [26]. The human skin originated from the excised skin panni obtained from female patients who underwent abdominoplasty, after they gave written consent (see Section 2.6). The experiments were conducted in agreement with the Declaration of Helsinki principles. The human skin for the ex vivo penetration studies was prepared as described in Section 2.6. The slices of human skin used in the skin penetration studies had a thickness of 1.10 to 1.30 mm. All DLs-in-chitosan hydrogel formulations (1.2 mL) were added in the donor chamber, whereas the receptor chamber was filled with 12.0 mL of Albunorm (5%, *v*/*v*) solution in PBS. As a control, curcumin in PG-in-hydrogel served as a control. The experiments were conducted for 8 h and sampling of the receptor medium (500 µL) was performed every hour. To maintain the sink conditions, the withdrawn receptor medium was replaced by an equal volume of fresh Albunorm^®^ (5%, *v*/*v*) solution in PBS. The penetrated curcumin was quantified as described in Section 2.2 using a standard curve of curcumin in Albunorm^®^/PBS (1:1, *v*/*v*). Experiments were performed in three replicates.

### 2.9. Statistical Analyses

To determine the level of significance (*p* < 0.05), statistical analyses were performed using one-way ANOVA test followed by Bonferroni’s multiple comparisons test performed on GraphPad Prism version 7.00 for Windows (GraphPad Software, La Jolla, CA, USA).

## 3. Results and Discussion

Chronic wounds exhibit severe molecular and cellular deficiencies and are heterogenous across the patient population; therefore, they are difficult to treat. At present, no wound dressing can be considered as “one size fits all”; however, there is a consensus in the field that chronic wound treatment should shift from conventional dressings towards precision medicine approaches [14]. It is known that the remodeling phase of wound healing comprises the deposition of extracellular matrix (ECM) in a well-defined network prior to wound contraction [3]. Chitosan promotes wound healing via stimulation of ECM formation as well as facilitating the mobilization, adhesion, and accumulation of red blood cells and platelets forming blood clots at the wound site [14]. Chitosan hydrogel could therefore be a perfect vehicle to incorporate bioactive molecules within nanoformulations. The combination of liposomal curcumin, assuring anti-inflammatory activity, and chitosan, providing wound healing and antimicrobial activities, could assure a synergistic effect and superior performance. The therapeutic potential of the novel dressing will be dependent on the characteristics of each component as well as their interplay.

### 3.1. Liposomal Characteristics

Considering the liposomes-in-hydrogel formulation, the liposomal size is known to influence both the hydrogel properties and the release of lipophilic compounds from liposomes-in-hydrogel systems [24]. Therefore, focusing on exploring the effect of the liposomal surface charge on both hydrogel properties and curcumin penetration through human skin, we aimed to prepare DLs of a similar vesicle size. The chosen vesicle size range was 200 to 300 nm, which has been suggested as appropriate for assuring dermal delivery via liposomes by offering the reservoir of the liposome-associated drug in the skin [29]. The extrusion method, utilized to reduce the vesicle size, allows the manufacturing of rather homogeneously distributed vesicles in the desired size range, as reported earlier [26]. For each liposomal dispersion, we adjusted both the number of extrusion cycles and membrane pore size, obtaining curcumin-containing DLs within the size range of 200 to 300 nm (Table 2), as targeted. Moreover, the vesicle size distribution was rather homogeneous for all DLs, as indicated by the rather low polydispersity.

DLs bearing different surface charges (Table 2) were obtained by including the selected surfactant in the liposomal phospholipid bilayers. NDLs comprised polysorbate 20 as a non-ionic surfactant, which conferred a neutral surface to DLs, as well as the neutral lipid, namely PC. Ionic surfactants were employed to obtain CDLs and ADLs, respectively. SA was responsible for the positive charge of CDLs, whereas SDCh conferred the negative charge to ADLs surface, both in agreement with the literature [30,31].

The entrapment efficiency of curcumin in the different DLs varied according to the liposomal composition (Table 2). The presence of surfactant might contribute to a lower entrapment of lipophilic compounds, such as curcumin; both substances are competing for accommodation within the liposomal phospholipid bilayer. Moreover, it is known that the drug/active substance incorporation capacity is additionally dependent on the type of employed surfactant [32]. In our case, ADLs exhibited the highest entrapment efficiency for curcumin. A possible explanation might be the higher lipophilicity of SDCh compared to polysorbate 20, thus improving the curcumin solubility and therefore enhancing the curcumin loading capacity for ADLs [32]. The presence of SA in CDLs has been shown to alter the lipid packing of liposomes by causing a charge repulsion [33]. This can therefore affect curcumin’s accommodation within the CDLs bilayer, as previously reported for the drugs of a similar lipophilicity as curcumin, such as celecoxib [34].

### 3.2. Effect of the Liposomal Surface Charge on the Texture Properties of Liposomes-In-Hydrogel

The development of effective hydrogel-based wound dressings requires their optimization in terms of hydrogel’s texture properties. For hydrogels as wound dressings, the hydrogel’s hardness can determine the ease of its application onto the skin, whereas the adhesiveness influences the hydrogel’s contact time with the skin. These factors consequently contribute to the efficacy of the treatment and assure patient compliance by reducing the frequencies of administration and the related pain caused by wound dressing changes [11]. While optimizing liposomes-in-hydrogel formulations, an evaluation of the texture properties of the hydrogel is required considering that the incorporation of DLs bearing different surface charges can affect the hydrogel texture properties [24]. Possible interactions between charged DLs and positive chitosan chains might change/affect the chitosan network, thus modifying its texture characteristics. The effect of the liposomal surface charge of curcumin-containing DLs on the hydrogel properties is presented in Table 3.

The incorporation of DLs negatively affected the original hydrogel texture properties (plain hydrogel). The incorporation of deformable liposomes weakened the hydrogel properties to a higher extent than the incorporation of free curcumin (CUR-in-hydrogel, Table 3). Due to the hydrophobic nature of curcumin, its incorporation in chitosan hydrogel in the form of free curcumin (not incorporated in DLs) was achieved by prior dispersion of curcumin in PG. The presence of PG has been shown to exert a positive effect on the viscoelastic properties of hydrogels by triggering hydrogen bonding between chitosan, water, and the solvent [35]. This might explain the positive effect of free curcumin in PG on the hydrogel properties compared to DLs. Vanić and co-workers [36] observed changes in the hydrogel texture properties when deformable propylene glycol liposomes were incorporated in Carbopol hydrogel; as in our case, the hydrogel texture properties were reduced. The incorporation of DLs in Carbopol hydrogel also affected the hydrogel texture when compared to a marketed gel product, although the authors did not focus on the effect of the liposomal surface charge [37]. Similar to our findings, Hurler and collaborators [24] also observed weakened chitosan hydrogel properties due to the incorporation of conventional liposomes bearing different surface charges. The charged DLs enhanced the hydrogel hardness while weakening both the cohesiveness and adhesiveness, as compared to NDLs (Table 3).

### 3.3. Effect of Liposomal Surface Charge on Hydrogel Bioadhesion to Human Skin

**Figure 1:**
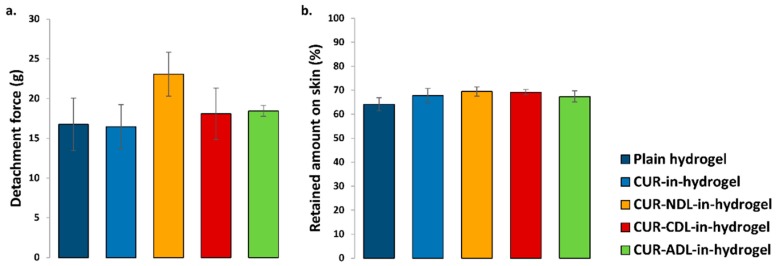
Effect of the liposomal surface charge on liposomes-in-hydrogel bioadhesiveness, expressed as the (**a**) detachment force and (**b**) amount of formulation retained onto the human skin surface. Results are expressed as mean (*n* = 3) ± SD.

Bioadhesiveness represents an important feature responsible for the effectiveness of hydrogel-based wound dressings. Good hydrogel bioadhesion to human skin can prolong the retention time of the incorporated drug/active substance at the skin site, thus resulting in an enhanced therapeutic effect. Considering the presence of the exudate in chronic wounds/burns, the contact time of the hydrogel to the skin might be reduced by a loss of the original hydrogel texture characteristics due to the high water content in the wounded area of exudating wounds [11]. Improved hydrogel bioadhesion to human skin might overcome this limitation. We therefore evaluated a possible effect of the liposomal surface charge on the chitosan hydrogel bioadhesiveness. Based on the method developed earlier in our laboratory [28], we evaluated the hydrogel bioadhesion to human skin by determining the force required to remove the hydrogel formulation from the skin (Figure 1a) and the amount of hydrogel formulations retained onto the skin surface at the end of the bioadhesion test (Figure 1b).

The bioadhesiveness expressed as the detachment force was not affected by the incorporation of curcumin-containing DLs, regardless of the liposomal surface charge. Both CDLs- and ADLs-in-hydrogel exhibited a similar bioadhesiveness as plain hydrogel, whereas NDLs-in-hydrogel showed the strongest bioadhesion to human skin. As observed for the texture properties, NDLs with a neutral surface seemed to stabilize the chitosan network to a higher extent compared to the charged DLs. Their neutral liposomal surface assures minimal interactions with the polymer, thus leaving unaltered the intrinsic bioadhesive properties of chitosan hydrogel.

The amount of the hydrogel retained on the skin was similar for all tested DLs-in-hydrogel systems. Moreover, the high amount of hydrogel retained onto the skin (approximately 70%) observed for all DLs-in-hydrogel is encouraging considering the need to potentially overcome the challenge of hydrogel exposure to wound exudate and reduced retention time when hydrogel is applied on exudate-rich wounded area. The high water content associated to those wound types is responsible for the limited adhesion of the wound dressing at the skin site. Therefore, the good bioadhesive properties of DLs-in-hydrogel systems confirmed herewith are promising in prolonging the retention time of novel dressing, including curcumin, at the wounded area rich in exudate, potentially enhancing its therapeutic outcome. Further studies involving the exposure to wound exudate are required to confirm these preliminary findings.

### 3.4. Stability of Hydrogel

**Figure 2:**
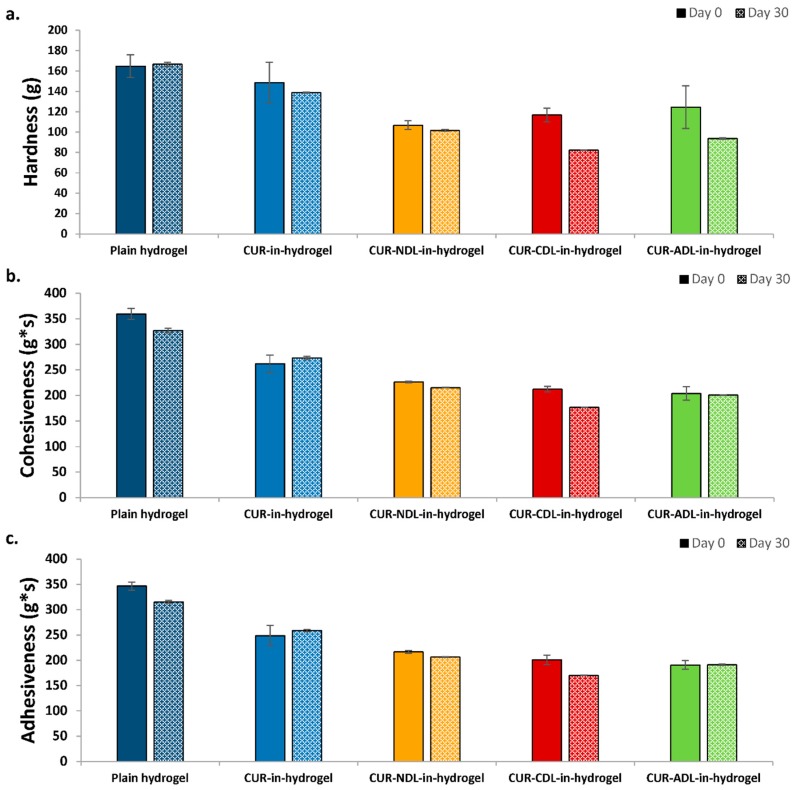
The effect of the liposomal surface charge on liposomes-in-hydrogel texture properties: stability testing. (**a**) hardness, (**b**) cohesiveness, and (**c**) adhesiveness, determined on day 0 and after storage at 23 to 24 °C for 30 days. Results are expressed as mean (*n* = 3) ± SD.

The incorporation of charged DLs in chitosan hydrogel has shown to influence, to a higher extent, the hydrogel’s texture properties (Table 2) and, to a lesser extent, the bioadhesiveness (Figure 1), possibly attributable to triggered interactions between charged DLs and chitosan. We therefore further explored the effect of the liposomal surface charge on the stability of liposomes-in-hydrogel. Wound dressings that can maintain their original properties when stored at room temperature would enable easier administration both by the patient itself or a caretaker. The liposomes-in-hydrogel formulations were stored at room temperature (23–24 °C) for one month and both the texture properties and bioadhesiveness of formulations were determined and compared with the properties of the original formulations. As expected, the original texture properties were preserved to a higher extent when NDLs were incorporated in the hydrogel compared to both CDLs and ADLs (Figure 2). The minimal interaction between neutral DLs and chitosan network seems to maintain the original hydrogel texture properties whereas the charged DLs weakened the texture properties due to electrostatic interactions. However, the findings were still considered acceptable, considering that the testing was performed at room temperature.

**Figure 3:**
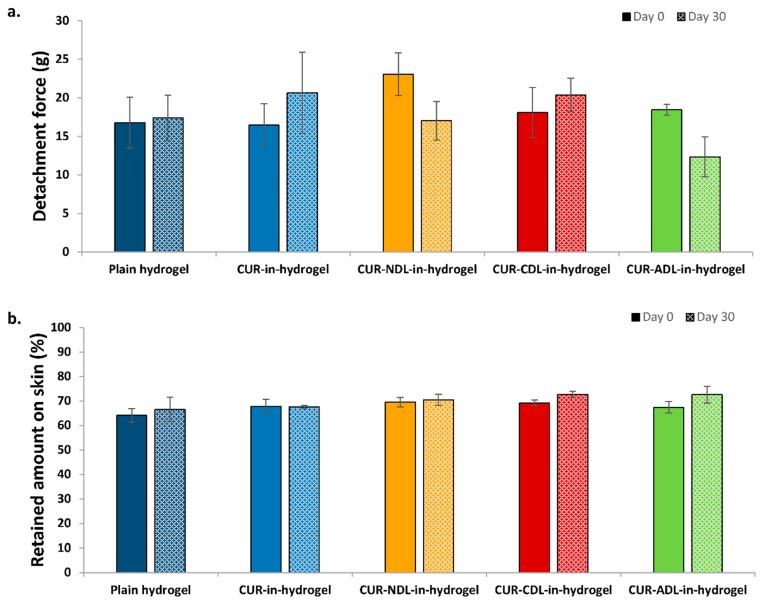
The effect of the liposomal surface charge on liposomes-in-hydrogel bioadhesiveness: stability testing. (**a**) Detachment force and (**b**) amount of formulation retained onto the skin surface, determined on day 0 and after the storage at 23 to 24 °C for 30 days. Results are expressed as mean (*n* = 3) ± SD.

Interestingly, the evaluation of the hydrogel’s stability in terms of its bioadhesiveness showed that the original bioadhesion to human skin was better preserved when CDLs were incorporated in the hydrogel (Figure 3). Both the detachment force (Figure 3a) and the amount of formulation retained onto the skin surface (Figure 3b) for CDLs-in-hydrogel moderately increased compared to both NDLs- and ADLs-in-chitosan hydrogel. However, the differences were not significant, and more experiments would be required to confirm the observed trend.

Although the original bioadhesiveness of liposomes-in-hydrogel was enhanced by the presence of NDLs, suggesting that the neutral surface of NDLs might exert a positive effect on hydrogel bioadhesiveness (Figure 1), the results from the hydrogel stability testing indicate that the positively charged DLs can better preserve hydrogel bioadhesiveness for a longer time as compared to NDLs. Considering the variability of human skin in respect to the source, the differences were not significant. The repulsion between positive CDLs and chitosan chains might stabilize the liposomes in the hydrogel matrix, thus preserving chitosan bioadhesion to human skin.

The bioadhesiveness of the dressing is one of the main factors determining the time curcumin is retained at the skin site. Despite the rather negative effect of charged liposomes on the texture properties of DLs-in-hydrogel, the incorporation of CDLs in hydrogel might increase hydrogel bioadhesiveness, thus assuring a longer time for curcumin to exert its biological activities. The significance of the superiority of CDLs in hydrogels needs to be confirmed. For comparison, visual evaluation of the hydrogels stored for one month indicated that the control, namely curcumin-in-hydrogel (non-liposomal curcumin in hydrogel), exhibited clear curcumin precipitation (data not shown). This was additional conformation that to deliver curcumin at the wounded site, combined formulations comprising liposomes and hydrogel are highly advantageous.

### 3.5. Ex Vivo Curcumin Penetration from DLs-In-Hydrogel into Human Skin

As dermal delivery systems, both DLs and chitosan hydrogel have shown ability to assure controlled and sustained skin penetration of the associated drug. When used in combination, this action can be enhanced and, together with the prolonged retention time at the targeted skin site, might lead to improved localized wound therapy [18,19]. Moreover, this can result in a reduction in frequency of wound dressings’ changes, improving patient compliance and the easiness of dressing administration. The incorporation of curcumin-DLs in the hydrogel introduces additional barriers that curcumin has to face and cross before reaching the targeted site. Therefore, curcumin release from the DLs-in-hydrogel followed by its skin penetration is a complex process and depends on several factors. The lipophilicity of curcumin will influence this process, especially its partitioning into the hydrogel, whereas its aqueous solubility will determine the diffusion rate through the hydrophilic hydrogel matrix, as reported for other lipophilic compounds [38]. The liposomal surface charge represents an additional factor that can affect curcumin penetration through/into the skin [39]. Moreover, due to the positively charged chitosan chains, the liposomal surface charge can determine possible interaction between DLs and chitosan [24]. This can affect the integrity of the liposomal bilayer when embedded into the hydrogel, thus influencing the release of curcumin. By exploring the abovementioned factors, the development of liposomes-in-hydrogel formulations for controlled dermal delivery of curcumin can be tailored and effective therapy assured.

**Figure 4:**
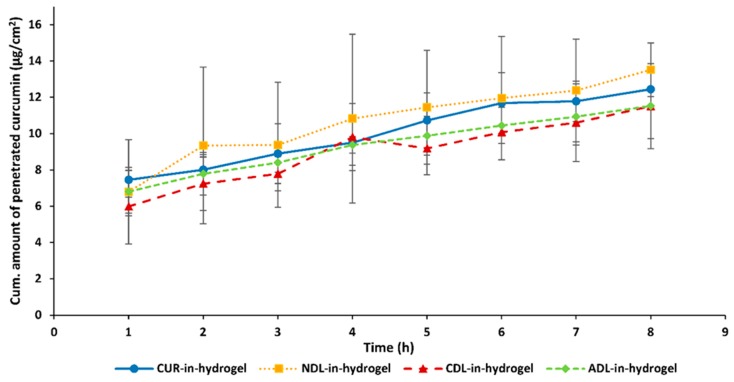
Ex vivo curcumin penetration from different DLs-in-hydrogel through full thickness human skin (*n* = 3 ± SD).

Figure 4 presents the skin penetration profiles of curcumin from the different curcumin-DLs-in-hydrogel systems. A minimal amount of penetrated curcumin was detected, regardless of the liposomal surface charge. This can be contributed to the slow diffusion of curcumin when released into the hydrophilic hydrogel due to its poor water solubility. In agreement with our findings, Jain and collaborators [25] also reported a rather slow in vitro release of curcumin from lipospheres embedded in hydroxyl propyl methyl cellulose-based gel.

As a control, curcumin-in-hydrogel was used; curcumin was incorporated in the hydrogel in a form of solution in PG in the same concentration as in DLs.

The NDLs-in-hydrogel release profile exhibited the highest amount of penetrated curcumin, even in comparison to curcumin-in-hydrogel (Figure 4). However, the differences were not statistically different and can only be considered as a trend. It is important to point out that the experiments were performed using human skin of different donors, adding to larger SD. NDLs with their neutral surface are not expected to interact with the chitosan chains. The minimal interaction between NDLs and chitosan chains might also marginally interfere with NDLs’ mobility within the polymer matrix, allowing faster curcumin delivery into/through the skin via NDLs. Thomas and collaborators [40] reported the enhancement of curcumin skin penetration from nanosystems-in-chitosan hydrogel compared to free curcumin-in-hydrogel. They incorporated curcumin in nanoemulsions further incorporated in chitosan hydrogel and observed a higher rat skin penetration of curcumin as compared to free curcumin-in-hydrogel. Sun and co-workers [41] reported higher penetration of curcumin from PLGA nanoparticles-loaded in Carbopol gel compared to free curcumin-in-gel, although they tested the formulations on mouse skin.

The charged DLs-in-hydrogel exhibited more sustained, although not significant, skin penetration of curcumin compared to NDLs-in-hydrogel and control (curcumin-in-hydrogel). Considering the possible electrostatic interactions that can be triggered when charged DLs are embedded into the chitosan network, the liposomal membrane might be more preserved, thus limiting curcumin partitioning into the hydrogel. The electrostatic interactions between negative DLs and positive chitosan chains can cause immobilization of ADLs within the hydrogel matrix, thus limiting initial curcumin diffusion into the hydrogel prior to reaching the skin. El Kechai and collaborators [42] proposed low mobility of liposomes bearing opposite charge to the hydrogel chains; the authors observed that positive liposomes were highly immobilized in the negative hyaluronic acid hydrogel. On the other hand, the higher repulsion occurring between CDLs and chitosan, both positively charged, might stabilize the CDLs membrane and limit curcumin partitioning into the chitosan hydrogel. Hurler and collaborators [24] also observed a similar release pattern when testing the in vitro release of a lipophilic compound from positively charged liposomes embedded in chitosan hydrogel, in agreement with our findings.

In our previous work [10], we showed a successful inhibition of *S. aureus* and *S. pyogenes* growth when testing curcumin in deformable liposomes in a curcumin concentration of 100 and 200 µg/mL. Furthermore, the curcumin-containing liposomes (even at a curcumin concentration of 2.5 µg/mL) successfully inhibited nitric oxide production. The concentrations used in the current study are in the same range as the ones in the aforementioned study, indicating that the delivery rate is compatible with the working concentration of curcumin in this type of carrier. Compared to Ternullo et al. [10], the ex vivo release through full thickness human skin exhibited a more sustained release when the DLs were incorporated into a hydrogel, as expected. Recently, Nguyen and coauthors [43] reported improved wound healing when utilizing a curcumin–oligochitosan nanoparticle complex and oligochitosan-coated curcumin-loaded liposomes with a curcumin concentration of 100 µg/mL. The hydrogel prepared in the current study contains curcumin in a concentration of 135 µg/g and is therefore similar to the concentration used by Nguen et al. (2019). The curcumin concentration in the novel dressing is sufficient to achieve the desired biological response.

Considering the prolonged retention time of curcumin assured by chitosan hydrogel and sustained delivery provided by liposomes-in-hydrogel, we can postulate that curcumin will have a sufficient time and required concentration at the skin site to exert its therapeutic effect. However, the findings should be further explored in a suitable wound model.

## 4. Conclusions

To utilize the wound healing potential of both curcumin and chitosan, we developed a novel wound dressing comprising curcumin-in-liposomes-in-chitosan hydrogel. Curcumin was incorporated in deformable liposomes of various surface charges and the effect of the liposomal surface charge on the performance of chitosan hydrogels was evaluated. Although the original texture properties were changed to a certain degree upon inclusion of the charged liposomes, the incorporation of DLS in the chitosan hydrogel did not affect its bioadhesiveness. Neutral deformable liposomes-in-hydrogel exhibited the strongest bioadhesiveness; however, their neutral surface did not preserve the hydrogels´s bioadhesion to human skin over longer storage times. The positively charged deformable liposomes exhibited an ability to stabilize the formulation’s bioadhesiveness and additionally assured sustained penetration of curcumin through the ex vivo full human skin. The developed advanced dermal delivery system is therefore promising as a wound dressing.

## Figures and Tables

**Table 1:** Liposomal composition.

Liposomes	Curcumin (mg/mL)	Lipoid S 100 (mg/mL)	Sodium Deoxycholate (mg/mL)	Stearylamine (mg/mL)	Polysorbate 20 (mg/mL)
NDLs	2.0	17.0	-	-	3.0
CDLs	2.0	15.3	-	1.7	3.0
ADLs	2.0	17.0	3.0	-	-

**Table 2:** Liposomal characteristics.

Liposomes	Diameter (nm)	Polydispersity Index	Zeta Potential (mV)	Curcumin/Lipid Ratio (µg/mg)	Entrapment Efficiency (%)
NDLs	288.73 ± 2.46	0.17 ± 0.02	–3.03 ± 0.24	51.54 ^a^ ± 0.99	43.97 ± 0.85
CDLs	252.24 ± 51.63	0.17 ± 0.01	34.01 ± 0.56	71.94 ± 2.32	55.04 ± 1.78
ADLs	291.54 ± 26.48	0.17 ± 0.01	–34.83 ± 0.64	89.67 ± 14.03	76.40 ± 11.96

Results are presented as mean (*n* = 3) ± SD. ^a^ Significantly lower vs. ADLs (*p* < 0.05).

**Table 3:** The effect of the liposomal surface charge on the texture properties of liposomes-in-hydrogel.

Chitosan Hydrogel Composition	Hardness (g)	Cohesiveness (g*s)	Adhesiveness (g*s)
Plain hydrogel	164.8 ± 11.1	359.6 ± 10.6	346.8 ± 8.0
CUR-in-hydrogel	148.7 ± 20.1	262.2 ± 17.0	248.5 ± 20.1
CUR-NDL-in-hydrogel	107.0 ^a^ ± 4.2	226.5 ^a,b^ ± 1.7	216.5 ^a^ ± 2.8
CUR-CDL-in-hydrogel	116.8 ^a^ ± 6.7	212.3 ^a,b^ ± 5.5	200.9 ^a,b^ ± 9.4
CUR-ADL-in-hydrogel	124.6 ± 21.0	204.0 ^a,b^ ± 13.5	190.7 ^a,b^ ± 8.6

Results are presented as mean (*n* = 3) ± SD. CUR: curcumin. ^a^ Significantly lower vs. plain hydrogel (*p* < 0.05); ^b^ significantly lower vs. CUR-in-hydrogel (*p* < 0.05).
